# Antiplasmodial Properties and Bioassay-Guided Fractionation of Ethyl Acetate Extracts from *Carica papaya* Leaves

**DOI:** 10.1155/2011/104954

**Published:** 2011-11-17

**Authors:** Paula Melariri, William Campbell, Paschal Etusim, Peter Smith

**Affiliations:** ^1^Division of Pharmacology, Department of Medicine, University of Cape Town Medical School K45, Old Main Building, Groote Schuur Hospital, Observatory, Cape Town 7925, South Africa; ^2^Department of Animal and Environmental Biology, Abia State University Uturu, PMB 2000, Abia State, Uturu, Nigeria

## Abstract

We investigated the antiplasmodial properties of crude extracts from *Carica papaya* leaves to trace the activity through bioassay-guided fractionation. The greatest antiplasmodial activity was observed in the ethyl acetate crude extract. *C. papaya* showed a high selectivity for *P. falciparum* against CHO cells with a selectivity index of 249.25 and 185.37 in the chloroquine-sensitive D10 and chloroquine-resistant DD2 strains, respectively. *Carica papaya* ethyl acetate extract was subjected to bioassay-guided fractionation to ascertain the most active fraction, which was purified and identified using high-pressure liquid chromatography (HPLC) and GC-MS (Gas chromatography-Mass spectrometry) methods. Linoleic and linolenic acids identified from the ethyl acetate fraction showed IC_50_ of 6.88 **μ**g/ml and 3.58 **μ**g/ml, respectively. The study demonstrated greater antiplasmodial activity of the crude ethyl acetate extract of *Carica papaya* leaves with an IC_50_ of 2.96 ± 0.14 **μ**g/ml when compared to the activity of the fractions and isolated compounds.

## 1. Introduction

*Carica papaya* L. of the family Caricaceae is a soft-stemmed perennial plant. It is usually unbranched and can grow to a height of about 20 m [1]. It is believed to originate from the Caribbean coast of Central America and over the years has found its way into many tropical and subtropical climates [2]. *C. papaya* can grow in male, female, or hermaphrodite forms. It is found growing wild in many parts of the tropics and is cultivated because of its sweet juicy fruit which serves as a nutritious food with rich medicinal value and also because of the ease with which it is digested. It commonly features in breakfasts, cooked in diverse ways, and as ingredients in jellies beverage and juice [3]. The fruits, leaves, and latex of this species are traditionally used in different parts of the world to treat diverse disease conditions. It is used in various places in the treatment of asthma, rheumatism, fever, diarrhea, boils, and hypertension and to increase the production of milk in lactating individuals [4]. Previous studies have shown that this species has promising antifungal [5], antibacterial [6], and anthelminthic [7] properties. However, studies on the *in vitro* antiplasmodial and cytotoxic properties of crude extracts sequentially extracted from solvents of different polarities are nonexistent in the literature. In this study, *Carica papaya* leaves were sequentially extracted with petroleum ether, dichloromethane, ethyl acetate, methanol, and water in that order. The antiplasmodial and cytotoxic activities of the extract from each solvent were investigated, and a bioassay-guided fractionation of the most active extract was carried out. 

## 2. Materials and Methods

### 2.1. Plant Materials

*Carica papaya* leaves were collected in June 2008 and identified by a taxonomist in the Plant Science and Biotechnology Department, Abia State University, Uturu, Nigeria. A voucher specimen PM/ABSU/06-63 of the plant was deposited in the herbarium of Abia State University, Uturu, Nigeria. The Division of Pharmacology at the University of Cape Town requested for the importation of these plants from Nigeria. The Nigerian custom services granted the demand of the Abia State University to export these materials to the University of Cape Town, South Africa for research purposes. 

### 2.2. Extraction

The air-dried leaves were reduced into smaller pieces using a plant blender (Waring, Conn, USA). Plants were sequentially extracted. This sequential extraction started with petroleum ether, which helps in reducing the chlorophyll pigment in these green leaves followed by dichloromethane extraction, ethyl acetate, methanol, and water. Each solvent was repeatedly used to extract each plant for 4-5 times. Plants were extracted for 24 hours, and during the process the plant material and the solvent were continuously shaken for adequate mixing on a horizontal orbit shaker (Labcon, Calif, USA). The resultant mixture was filtered and the filtrate concentrated under pressure in a Büchi Rotavapor R-205 (Büchi Labortechnik AG, Switzerland), at 24°C. The concentrated extracts were transferred to preweighed vials, dried in the hood at room temperature, and stored at −20°C until used. The water extracts were concentrated by freeze drying using a DURA-DRY II instrument (FTS Systems, NY, USA) under a reduced pressure at −82°C. The freeze-dried extracts were stored at −20°C. The extractive value (% w/w) of the dry extracts was 23.4%. 

### 2.3. Parasite

The chloroquine-sensitive strain (D10) which was used for this experiment was donated by Dr. A. Cowman, Walter and Eliza Hall Institute of Medical Research, Melbourne, Australia, while the chloroquine resistant strain (DD2) was derived from Indochina. The asexual erythrocytic stages of these parasites were maintained in a continuous culture using the method of Trager and Jensen [8]. 

### 2.4. *In Vitro* Experimental Plate Setup and Antiplasmodial Assay Procedure

The antiplasmodial activity of each extract was evaluated against the chloroquine-sensitive (CQS D10) and chloroquine-resistant (CQR) strains of *P. falciparum *using the parasite lactate dehydrogenase (pLDH) assay as described by Makler et al. [9]. A stock solution of 2 mg/mL of extract was prepared in 10% methanol (MeOH) (in deionized water). This was further diluted in complete medium to attain a final concentration of 200 *μ*g/mL in 1% MeOH. The stock solutions were prepared on the assay day. Chloroquine (CQ) (Sigma) was used as the standard reference drug (positive control). 2 mg/mL stock of CQ (Sigma) was constituted in deionized water and further diluted in complete medium to a concentration of 200 *μ*g/mL. Extracts were serially diluted twofold in complete medium up to 0.195 *μ*g/mL using a flat-bottomed, 96-well microtitre plate (Greiner Bio-One). CQ was tested at a starting concentration of 100 ng/mL or 1000 ng/mL for the sensitive (D10) and resistant (DD2) strains of *P*. *falciparum*, respectively. Unparasitised erythrocyte (RBC) was added to column 1 (blank) which had no drugs, while parasitized red blood cells (pRBCs) were added to columns 2–12. The plate was gassed for 2 minutes (93% N_2_, 4% CO_2_, and 3% O_2_) and incubated for 48 hours. A final hematocrit and parasitemia of 2% was used for all experiments. The IC_50_ recorded in this study with the exception of the screening assay is the mean of 3 independent experiments. The absorbance of each well was read using a microplate reader at 590 nm. The percentage parasite survival and the concentration that inhibits the growth of parasites by 50% were determined by measuring the conversion of NBT by *P. falciparum*. This was achieved by analyzing the readings from the microplate reader using Microsoft Excel 2002, and the IC_50_ value which is the concentration at which the growth of the parasite was inhibited by 50% was determined using a nonlinear dose response curve fitting analysis in Graph Pad Prism version 4. 

### 2.5. *In Vitro* Cytotoxicity Assay

The cytotoxicity assay used in this study was the method described by Mosmann et al. [10]. This is a rapid colorimetric assay method for determining cellular growth and chemosensitivity. It makes use of 3-(4,5-dimethylthiazol-2-yl)-2,5-diphenyltetrazolium bromide (MTT) salt. The mammalian Chinese hamster ovarian (CHO) cell line was used to investigate cytotoxicity activity. Emetine (Sigma) was used as the standard reference drug (control) to establish the cytotoxicity of the sample against the CHO cell lines. Two mg/mL stock of emetine was constituted in deionised water and stored at 4°C. A 10% dilution of stock solution in CM was made in complete medium to give the highest concentration of 200 *μ*g/mL and the lowest concentration of 0.002 *μ*g/mL. These concentrations were tested to determine the cytotoxicity of test samples against the CHO cells, as well as the IC_50_ by comparing with the reference drug emetine. The cell survival was determined using a microplate reader at 540 nm wavelength. The data was analyzed using Microsoft Excel 2002 and Graph Pad Prism; version 4 was used for the nonlinear dose response curve analysis. The IC_50_ values were given as a mean of 2 or more independent experiments. The selectivity index (SI), which is the cytotoxicity: antiplasmodial ratio, was calculated to determine if the recorded activity was due to the antiplasmodial activity of the test samples or due to cytotoxicity to CHO cells. The resistance index (RI) for a drug is the ratio between the IC_50_ of the resistant values of a strain to the sensitive strain. The selectivity index (SI) and the resistance index (RI) were calculated as shown below:

SI = IC_50_ cytotoxicity/IC_50_ antiplasmodial activity,
RI = IC_50_ of resistant strain (DD2)/IC_50_ of sensitive strain (D10).

### 2.6. Bioassay-Guided Fractionation of *C. papaya* Ethyl Acetate Fraction

#### 2.6.1. Solid-Phase Extraction (SPE) Procedure

The ethyl acetate extract which gave the most antiplasmodial activity was fractionated using the solid-phase extraction (SPE) procedure. This was carried out with reverse-phase octadecyl C_18_ Isolute cartridges (2.6 × 13.0 cm, 10 g sorbent, IST Ltd, Anatech, South Africa). Samples of the ethyl acetate extract from the leaves of *C*. *papaya* were dissolved in methanol. This mixture was diluted to a concentration of 5 mg/mL with a 60% acetonitrile concentration in water (60% ACN : 40% mH_2_O). The C_18_ isolute cartridge was premoistened with 20 mL of mH_2_O and preconditioned with 20 mL of the 60% acetonitrile. A volume of 3 mL of this solution was layered on the top of this cartridge. Samples retained on the sorbent beds were rinsed with 20 mL of mH_2_O to elute unretained material. Samples retained on the sorbent beds were eluted under vacuum through a step-wise gradient with 40 mL of ACN : H_2_O (20%–100%) at an increasing concentration of 20%. The eluates were collected in a fitted bottle. The vacuum pressure was set to control the flow at the rate of 15 mL/min. At the end of each run, the cartridge was washed with 100 mL acetone to wash out any remaining material. The collected fractions were concentrated under pressure by rotary evaporation at 40°C and freeze-dried. The freeze-dried samples were placed in vials and stored at −20°C. The 100% ACN fraction was transferred to preweighed vials, dried in the fume hood, and stored at −20°C. The *in vitro* antiplasmodial activities of these fractions were determined using the method described earlier in Section 2.4.

#### 2.6.2. High-Pressure Liquid Chromatography (HPLC)

The SPE fraction selected for further purification was fractionated on a Shimadzu LC 10AS high-pressure gradient system. This was equipped with a desktop PC which runs Shimadzu control software via a Shimadzu CBM10A communication bus module. Other components of the HPLC instrument included an automatic sample injector, two solvent delivery systems (LC10AS pumps), and a diode array detector (Shimadzu SPDM10A). Compounds were detected by UV spectra at 210 nm, 240 nm, and 260 nm as acquired by the diode array detector. The solvents used include methanol (Scharlau) and acetonitrile (Scharlau), each of analytical grade. Purified deionized water (Millipore, milli-Q water system) was also used. The conditions are stated in Section 2.6.3. 

#### 2.6.3. Semipreparative HPLC Conditions

A semipreparative HPLC C_18_ column (Discovery, 25 cm × 10 mm, 5 *μ*m, 56924-U Supelco) was used. Samples were chosen based on their *in vitro* antiplasmodial activity, as well as their cytotoxicity values. Samples were centrifuged in a microcentrifuge (Abbott, Germany) at 1000 rpm in 5 minutes. The injection volume was 50 *μ*L with a flow rate of 2 mL/min over a 30 min run time and a solvent gradient of 20–100% acetonitrile in water. The elution time of the peaks was observed, noted, and set up for further collections following multiple injections. The fractions collected were concentrated using the rotary evaporator and freeze-dried. Dried samples were tested *in vitro* against *Plasmodium* parasites, and the active peaks were tested for purity using an analytical HPLC and GC-MS systems as specified in Section 2.6.4. 

#### 2.6.4. Analytical HPLC and GC-MS Conditions

The purity of the peaks was monitored by an analytical HPLC using an octadecyl Silica column (Agilent Eclipse, XDB-C_18_, 4.6 × 150 mm, 5 *μ*m, USA). Separations were accomplished at 29.7°C with a solvent gradient of 20–100% acetonitrile in water for 30 minutes at a flow rate of 1 mL/min. The purity and identity of these peaks were further confirmed using the GC-MS spectrometry. The carrier gas was helium with a constant flow of 1 mL/min. The injection split was 1 : 5; the temperature of the injector and the transfer temperature were 280°C. The EI ionization energy was 70 eV, and the scanning mass range was *m/z* 40 to 400 (perfluoro-tri-*N*-butylamine as mass reference), with a solvent hold of 6 minutes. 

## 3. Results

### 3.1. *In Vitro* Assay

The results of the screening assay showed that the highest antiplasmodial activity was found in the ethyl acetate (EA) fraction using the chloroquine-sensitive D10 strain, with IC_50_ value of 2.6 *μ*g/mL when compared to other solvents (Table 1). In this study, *in vitro* activities of ≤10 *μ*g/mL were regarded as active; thus, work with the other extracts was not taken further. Chloroquine used during this screening showed an IC_50_ of 8.55 ± 2.81 ng/mL in the CQS D10 strain. The growth of the parasites treated with the ethyl acetate extract was significantly inhibited. Water extracts had no effect on the growth of the parasite.

The *in vitro *antiplasmodial and cytotoxicity activities of the ethyl acetate extract are shown in Table 2. The ethyl acetate fraction of *C. papaya *showed a high selectivity for *P. falciparum *with a selectivity index of 249.25 and 185.37 against the D10 and DD2 strains, respectively (Table 2). The D10 strain used in the experiment was found to be CQ-sensitive with 50% inhibitory concentration (IC_50_) value of 9.21 ± 3.01 ng/mL, while the DD2 strain showed IC_50_ value of 98.5 ± 26.1 ng/mL. Emetine recorded IC_50_ of 0.045 *μ*g/mL.

### 3.2. Solid-Phase Extraction (SPE)

Fractionation of the ethyl acetate fraction of *C. papaya* by solid-phase extraction was carried out to isolate and identify the active components (Section 2.6.1). 900 mg of ethyl acetate extract was fractionated using the solid-phase extraction (SPE) procedure. Weights of the different fractions and their activity against the D10 strain of *P. falciparum *are shown in Table 3. The activity was greatest in the more hydrophobic fractions. 

### 3.3. High-Pressure Liquid Chromatography (HPLC)

**Figure 1:**
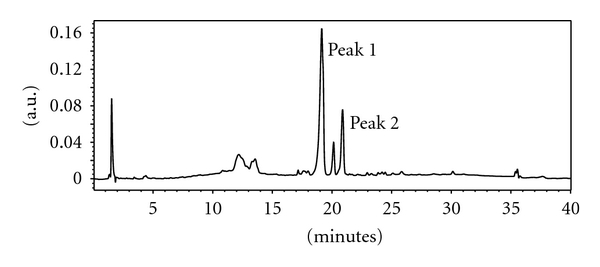
HPLC profiles of SPE fraction using C_18_ column: agilent XDB C_18_ RP analytical: 4.6 × 150 mm; 5 *μ* particle size (*λ* = 236.8 nm). HPLC conditions: mobile phase ACN: H_2_O using a gradient of 20–100% ACN (30 minutes) 100% ACN hold (3 minutes), 100-20% ACN (2 minutes) 20% ACN hold (5 minutes), Injection volume: 30 *μ*L of 1 mg/mL, column temp.: 30°C, flow rate: 1 mL/min.

The HPLC profile of the *C. papaya* ethyl acetate SPE fraction (100% ACN) showed two major peaks. These were isolated using semipreparative HPLC column. The 100% ACN fraction was further fractionated using HPLC analytical system and revealed the chromatogram shown in Figure 1.

The recorded activity of the 100% ACN fraction was very close to the activity of the parent ethyl acetate crude extract of 2.96 *μ*g/mL against *P. falciparum* (Table 2). Further purification of the 100% ACN fraction using an analytical HPLC column yielded two major peaks. Peak 1 had IC_50_s of 3.58 *μ*g/mL and 4.40 *μ*g/mL against the CQS D10 and CQR DD2 of *P. falciparum*, respectively, while peak 2 recorded IC_50_ values of 6.88 *μ*g/mL and 6.80 *μ*g/mL against the CQS and CQR strains of *P. falciparum*, respectively (Table 4). These two peaks were less active than the SPE fraction (2.2 *μ*g/mL) shown in Table 3 as well as the ethyl acetate extracts which had an IC_50_ of 2.96 *μ*g/mL against the CQS strain and 3.98 *μ*g/mL against the CQR strain (Table 2). 

### 3.4. GC-MS Analysis

**Figure 2:**
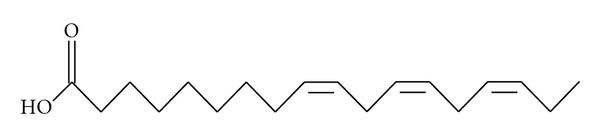
9,12,15-octadecatrienoic acid (linolenic acid).

**Figure 3:**
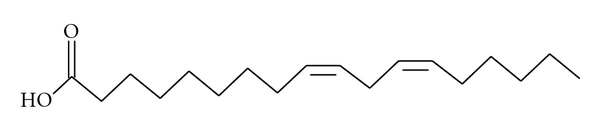
9,12-octadecadienoic acid (linoleic acid).

Peaks 1 and 2 were identified as essential fatty acids 9,12,15-octadecatrienoic acid (linolenic acid) and 9,12-octadecadienoic acid (linoleic acid), respectively, (Figures 2 and 3) using the GC-MS spectrometry. These essential fatty acids belonging to the C_18_ fatty acid differ structurally in the position and degree of unsaturation. 

An attempt was made to characterize and elucidate the structures of compounds 1 and 2 using the 1D and 2D NMR spectrometric methods. The ^1^H and ^13^C spectra used in this study are the most widely used 1D NMR techniques. ^1^H–NMR spectra can identify the protons in molecules. The number of ^13^C signals identified compounds 1 and 2 as unsaturated aliphatic fatty acids. Generally, 1D NMR helps in identification of aliphatic systems and determination of the degree of unsaturation, as well as the identification of functional groups. Further characterization of compounds 1 and 2 using 2-D NMR techniques which included HSQC, HMQC, and gCOSY met with difficulties due to the similarity in chemical shift of most of the methylene groups and of the olefinic double bonds. However, two groups could be unequivocally identified. The CH_2_ at position 2 gave a triplet at *δ*2.30, with a coupling constant of 7.50 Hz. Similarly the CH_3_ at position 18 gave a triplet at *δ*0.90 with a coupling constant of 3.6 Hz. The multiple peaks for compound 1, which were at ^1^H 5.27–5.38 (9, 12, 15 H), were connected to carbon signals at ^13^C 131.0–127.0 in the HSQC spectrum, while in compound 2 the multiple peaks at ^1^H peaks, which were at *δ*H 5.30–5.39 (9, 12 H) were linked to the carbon signals at ^13^C 129.0–130.6 in the HSQC spectrum. Due to the complexity of the resonances for the olefinic protons in a similar chemical environment, the purity of these compounds was further confirmed using the GC-MS spectrometry. Gas chromatography is routinely used to analyze fatty acids due to its high resolution, speed, and sensitivity. The GC-MS spectrum of compound 1 shows the molecular ion at *m/z* 278. In compound 1 two losses of CH_2_ groups were evident (*m/z* 135–*m/z* 121; *m/z* 93–79), while in compound 2 the molecular ion was shown at *m/z* 280. Two losses of CH_2_ groups were also evident (*m/z* 96–*m/z* 82; *m/z* 82–67) in the spectrum of compound 2.

## 4. Discussion

In this study antiplasmodial activities of ≤10 *μ*g/mL were regarded as active. According to Gessler et al. [11] very good extracts should display IC_50_s of ≤10 *μ*g/mL. Water extracts showed no activity with IC_50_ values >50 *μ*g/mL. Irungu et al. [12] demonstrated similar results in work with 14 plants. Bhat and Surolia [13] recorded no activity of the water extracts of *C. papaya. *The petroleum ether extracts of the rind and pulp of the unripe fruit of *C. papaya* demonstrated antiplasmodial activities with IC_50_ values of 15.19 *μ*g/mL and 18.09 *μ*g/mL, respectively [13]. Their observations using FCK 2 (a local strain of *P. falciparum* from Karnataka state, India) were similar to the IC_50_ value of 16.36 *μ*g/mL from the petroleum ether extracts of the leaves of *C. papaya *investigated in this study using the D10 strain of *P. falciparum.*

In the present study, *C. papaya* ethyl acetate extract showed a high selectivity 249 and 185 against *P. falciparum*-sensitive (D10) and *P. falciparum*-resistant (DD2) strains, respectively. This indicates good specificity against *P. falciparum* and also shows that the recorded activity of the *C. papaya* extract was not due to a nonspecific cytotoxic effect. In general, an SI ≥10 signifies that biological efficacy is not the result of *in vitro* cytotoxicity [14, 15]. The activity of the ethyl acetate extracts against the chloroquine-sensitive (D10) and chloroquine-resistant (DD2) strains of *P. falciparum* did not differ significantly. The control drug for the *in vitro* antiplasmodial experiment was chloroquine, while that for cytotoxicity experiment was emetine [15–17]. Previous studies have shown that *C. papaya* ameliorates vaginal disturbances due to *Trichomonas vaginalis *[18]. It has been reported to show anti-inflammatory properties [19]. The anthelminthic activity of *C. papaya* is traceable to the presence of carpain (alkaloid), carpasemine (benzylthiourea), and benzylisothiocyanate [20]. The latex of *C. papaya* at a dose of 8 g/kg has been found to be 84.5% effective against *Heligmosomoides polygyrus* (in mice) and *Ascaris suum* in pigs [7]. These researchers reported a dose response activity of papaya latex and stated that the calculated ED_100_ of papaya latex against adult *Heligmosomoides polygyrus* was 12 g/kg using probit analysis [7]. A previous study demonstrated the potency and cost effectiveness of *C. papaya* fruit when applied topically in the treatment of chronic ulcers in Jamaica [21]. In a recent study, *C. papaya *was listed as one of the plants used in the treatment of leishmaniasis [22]. It has been reported as accessible, nontoxic, and prophylactic and to be a promising monotherapy against intestinal parasitosis in tropical countries [23]. 

The activity of the ethyl acetate extract of *C. papaya* was stronger than that of the isolated compounds in this study. This observation suggests that the various compounds in the mixture may act synergistically. Neither peak showed significant cytotoxicity. In this study, the *in vitro* activity of linolenic acid which has three double bonds was higher than linoleic acid which has two double bonds. There was no significant difference in the activity of these compounds in the D10 and DD2 strains used in this study. The antiplasmodial activity of the unsaturated fatty acids has been reported to increase as the degree of unsaturation increases [24, 25]. These researchers reported the marked *in vitro* growth inhibition of *P. falciparum* by docosahexaenoic acid (C_22-6,n-3_), docosahexaenoic acid methyl ester (C_22-6,n-3_ methyl ester), eicosapentaenoic acid (C_20-5,n-3_), arachidonic acid (C_20-4,n-6_), and linoleic acid (C_18-2,n-6_). They reported that oleic acid (C_18-1,n-9_) and docosanoic acid (C_22-0_) had very little effect on parasite growth inhibition [24]. In their work, the unsaturated fatty acids C_22:6,n-3_ and C_20:4,n-6_ showed significant *in vitro* antiplasmodial activity but C_22:0_ was inactive. 

The introduction of a single double bond into the mono-unsaturated fatty acid greatly enhanced the antiplasmodial effects of the molecules [24]. Further work on a C_18_ fatty acid (scleropyric acid) isolated from the twigs of *Scleropyrum wallichianum* Arn. of the family Santalaceae Suksamrarn et al. [26] reported antiplasmodial activity with an IC_50_ value of 7.2 *μ*g/mL against K1 (CQR) strain of *P. falciparum*, similar to the antiplasmodial activity of linoleic acid with an IC_50_ of 6.80 *μ*g/mL against the DD2 (CQR) strain in this study. Further study documented the antiplasmodial activities with IC_50_ < 5 *μ*g/mL showed by fatty acids isolated from *Croton lobatus* against *Plasmodium falciparum *K1 (CQR) strain [14]. The fatty acids they isolated included (*Z*.,*Z*.,*Z*.)-9,12,15-octadecatrienoic acid methyl ester, 8,11,17,21-tetramethyl-(*E*.,*E*.,*E*.,*E*.)-8,10,17,21-tetraentetracosanoic acid, (*E*.)-3-(4-methoxy-phenyl)-2-phenyl-acrylic acid, and betulinic acid [14]. A previous study reported that the neutrophil-mediated killing of the asexual blood forms of *Plasmodium falciparum* could be enhanced by fatty acids [25]. These essential fatty acids are recently used as health supplements due to the health benefits associated with them [27, 28]. The lipophilic nature of these acids which were the active components isolated from *C. papaya* ethyl acetate extract in the present study may help explain the poor activity exhibited by the aqueous extract in a recent study [29]. 

 In conclusion, ethyl acetate fraction of *C. papaya* demonstrated the greatest antiplasmodial activity when compared to the activities of the SPE fractions and the isolated compounds. This suggests an enhancement of activity by other chemical constituents present in the extract which may have acted synergistically. The hot water extraction of these plants used by the traditional healers could extract lower concentrations of these active lipophilic components, but may not be available at therapeutic doses. This result may help explain the increase in parasite survival despite continuous treatment with herbal remedies. Hot water extracts of plants can be difficult to evaluate for antiplasmodial activity as they can contain large amounts of saponins which have nonspecific antiplasmodial activity. An investigation of the *in vivo* schizontocidal activity of the fractions is necessary since *in vitro *activity does not mean that the chemical compound is equally active *in vivo* [30]. This is because some physiological factors and immune response that are inevitable in an *in vivo* system are not applicable in the *in vitro* experiment. 

## Figures and Tables

**Table 1:** The *in vitro* antiplasmodial activity of *Carica papaya* leaves extracted with the various solvents using the CQS D10 strain.

Plant botanical name	PET IC_50_ (*μ*g/mL)	DCM IC_50_ (*μ*g/mL)	EA IC_50_ (*μ*g/mL)	MEOH IC_50_ (*μ*g/mL)	H_2_O IC_50_ (*μ*g/mL)
*C. papaya*	16.4	12.8	2.6	10.8	>50.0

PET: petroleum ether; DCM: dichloromethane; EA: ethyl acetate; MEOH: methano; H_2_O: water.

**Table 2:** *In vitro* antiplasmodial activity of *Carica papaya* on *Plasmodium falciparum *cultures and toxicity towards the CHO cell line.

Crude extract	Solvent	IC_50_ D10 (*μ*g/mL)	IC_50_ DD2 (*μ*g/mL)	IC_50_ CHO (*μ*g/mL)	(SI) D10	(SI) DD2	RI
*Carica papaya*	EA	2.96 ± 0.14	3.98 ± 0.42	737.8 ± 0.28	249.25	185.37	1.34

SI = IC_50_ cytotoxicity/IC_50_ antiplasmodial activity. RI = IC_50_ of resistant strain (DD2)/IC_50_ of sensitive strain (D10).

**Table 3:** Activities of *C. papaya* SPE fractions against the CQS D10 strain.

Percentage ACN fraction	Weight of Fractions (mg)	IC_50_ (*μ*g/mL)
20%	142.80	>50
40%	295.60	16.55 ± 3.09
60%	329.90	2.52 ± 0.39
80%	482.51	2.69 ± 0.76
100%	369.50	2.24 ± 0.58

**Table 4:** *In vitro* activity of peaks 1 and 2.

Sample name	D10 IC_50_ (*μ*g/mL)	DD2 IC_50_ (*μ*g/mL)	CHO IC_50_ (*μ*g/mL)	SI-D10 CHO	SI-DD2 CHO	RI
Peak 1	3.58 ± 0.22	4.40 ± 1.10	54.70 ± 2.01	15.27	12.43	1.22
Peak 2	6.88 ± 0.02	6.80 ± 1.21	51.16 ± 5.52	7.43	7.52	0.98

SI = IC_50_ cytotoxicity/IC_50_ antiplasmodial activity. RI = IC_50_ of resistant strain (DD2)/IC_50_ of sensitive strain (D10).
